# Estimation of age in forensic anthropology: historical perspective and recent methodological advances

**DOI:** 10.1080/20961790.2018.1549711

**Published:** 2019-03-19

**Authors:** Douglas H. Ubelaker, Haley Khosrowshahi

**Affiliations:** Department of Anthropology, Smithsonian Institution, Washington, DC, USA

**Keywords:** Age estimation, forensic sciences, forensic anthropology, history, recent advances

## Abstract

Estimation of age represents a central focus of forensic anthropological analysis of human skeletal remains and of the living. Advances registered in recent research include the topics of taphonomic impact, new anatomical areas of interest, histology, population variation, the dental pulp chamber, technology, mathematical approaches, biochemical analysis and techniques specifically targeting the living. This article reviews the historical development of age estimation methods and considers likely future directions.

## Introduction

Estimation of age represents one of the most important aspects of analysis in forensic anthropology. Of the different variables within the biological profile of missing persons represented by recovered skeletal remains, age at death represents a key feature leading to identification [1–3]. Selection of appropriate methods depends of course on what skeletal elements are present and what general age is represented. Techniques utilized to estimate age at death in fetal, infant, child and other immature individuals differ from those needed to analyse the skeleton from a mature individual [4,5]. If the preliminary assessment of recovered remains reveals that the third molar is completely formed, the epiphyses of the iliac crest and sternal clavicle are united, and the basilar synchondrosis is fused, then techniques appropriate for mature individuals should be employed. These epiphyses are the last to fuse. Thus if they are fused, those of the other long bones (distal femur, distal radius proximal tibia, etc.) normally are fused as well.

Methods for adult age estimation are extensive but include assessment of the extent of cranial suture closure, parietal thinning, pubic symphysis metamorphosis, development of the sternal rib ends, osteoarthritis including osteophytosis, overall degenerative changes, changes to the auricular area and acetabulum of the pelvis, as well as dental and bone histology features. This article examines recent methodological advances in these techniques to assess age from the human skeleton and in the living. The intent is not to summarize all available methods and their individual histories but rather to focus on the historical developments and recent advances in age estimation methods as documented in the published literature. Greater detail on the methods and issues is available in the literature cited.

## Age estimation of the immature

Regarding immature individuals, research and casework have demonstrated that some age information is available from bone size and maturation, epiphyseal appearance and closure, loss of deciduous teeth and eruption of both deciduous and permanent teeth. However, the most accurate estimates are those generated from an assessment of dental formation when teeth are available for examination. Significant advancements in age assessment of dental formation involve the evaluation of individual teeth, utilizing local standards and recognition of sex and population differences. Although many standards are available, advancement consists of utilizing a system that is locally-based, defines the various stages of dental formation and provides age information based upon an extensive, appropriate study sample.

Assessment of epiphyseal closure also must consider a substantial sex difference and population variation; note however that some error is involved in sex estimation. Considerable time is required from the beginning to the end of the closure. Thus, the use of standards must carefully examine the definition of “closure” employed in each particular study. Since population variation exists in the timing of epiphyseal closure, it is crucial to choose standards appropriate for the individual examined. Nutritional status and related socioeconomic factors can affect the aging process.

## Historical development of methods

Methods for estimating age in adults have changed dramatically throughout the history of forensic anthropology. In general, the evolution of methods increasingly recognizes the value of using multiple methods/age indicators over single ones and that those techniques developed from one study sample may not be applied to others with the same level of confidence. For example, as early as 1955, Brooks examined age indicators in the cranium and pubis noting considerable sex differences and varying reliability. In this early study, Brooks [6] concluded, “no one age indicator is adequate”. The value in examining multiple age indicators was also emphasized in the later classic texts by Krogman [7] and Stewart [8].

Controversy in the paleodemography literature stimulated subsequent advancements in age at death estimation methodology. In 1982, Bocquet-Appel and Masset [9] published an article “Farewell to paleodemography” criticizing the accuracy of this endeavor. While some concern was aimed at sampling problems relating to ancient cemetery excavation, most criticisms focused on the methods of age estimation. Concerning estimates of adult age at death, the publication argued that the age structure of the reference sample influenced age estimates for the target skeletons. Of course, each published method of individual techniques was based on a particular reference sample, sometimes with limited age distribution. Bocquet-Appel and Masset [9] argued that this had a devastating effect on the general reconstruction of demographic variables and individual age estimates in particular. Others noted that while Bocquet-Appel and Masset had raised legitimate issues, they had exaggerated the magnitude of concern. This point was made clear in a follow-up article by Van Gerven and Armelagos [10] “‘Farewell to paleodemoraphy’ rumors of its death have been greatly exaggerated.” Bocquet-Appel and Masset [11] remained unconvinced.

The exchange cited above was followed by substantial research and discussion both within the fields of paleodemography and forensic anthropology. In 1983, Meindl et al. [12] critically examined accuracy in age estimation methods. Later, in 1985, Meindl and Lovejoy [13] produced new data on ectocranial suture closure, including a new system for scoring closure. Also in 1985, Meindl et al. [14] offered a revision of age estimation from the os pubis with a critical look at other methods involving that bone. Lovejoy et al. [15] also examined age changes in the auricular surface of the ilium, offering a new approach for that area of the pelvis. In 1985, Lovejoy et al. [16] returned to the theme advanced earlier that combined (multifactorial) methods are more accurate than single ones.

In the 1990’s publications began to appear that compared results of different methods, including those using the same area of the skeleton. For example, Brooks and Suchey [17] evaluated age estimates based on two methods that involved examination of the os pubis. Konigsberg and Frankenberg [18] examined the uncertainty included in age estimates. Bedford et al. [19] tested the multifactorial aging approach on skeletons of known age from a Canadian collection. Hershkovitz et al. [20] examined the factors limiting the accuracy of age estimates from the sagittal suture. Also in 1997, Aykroyd et al. [21] discussed the methodology of regression analysis in adult age estimation. Belkin et al. [22] noted the impact of environmental factors on the aging process. Galera et al. [23] compared the error involved when different methods of assessment of cranial suture closure were applied to the same collection (Terry at the Smithsonian Institution in Washington, DC).

In 1999, Baccino et al. [24] published a study in which seven methods of assessing age at death were applied to a single French collection of individuals of known age at death. In a blind study, they applied the dental Lamendin method [25], the Suchey-Brooks method of assessing the pubic symphysis [17], the İşcan method relating to the sternal rib ends [26], the Kerley histological method [27] and three combined methods. All of the combined methods outperformed any of the individual techniques. The accuracy of the latter reflected the experience of the investigator.

In 2000, Milner et al. [28] summarized state of the art regarding age estimation in paleodemography studies, noting the continued challenge of accurate age estimation. Hoppa [29] called attention to population variation impacting age estimation. Discussion of age estimation in paleodemography continued to evolve [18]. Also in 2002, Wittwer-Backofen and Buba [30] introduced a new method of age estimation involving assessment of tooth cementum annulation, a promising microscopic method but with problems of feature identification contributing to interobserver error.

In 2002, Boldsen et al. [31] introduced transition analysis as a new approach to age estimation. Transition analysis represented a response to the Bocquet-Appel and Masset [9] reference sample issue, as well as concerns expressed by Kemkes-Grottenthaler [32] and Hoppa and Vaupel [33]. It featured a multifactorial approach based on multiple study collections that generated a most likely age estimate along with a sense of the probabilities involved.

In 2006, Bello et al. [34] again called attention to age and sex bias in paleodemographic reconstruction. Rinaldi et al. [35] critically examined methodology involving cranial suture closure. Frederic et al. [36] took a fresh look at Bayesian statistics in age estimation using tooth wear assessment. Ardito and Pacciani [37] also highlighted Bayesian approaches to age estimation. Milner et al. [38], Garvin et al. [39] and Milner and Boldsen [40] all provided reviews of new methodology in age estimation at that time.

In more recent years, tests have emerged focusing on multifactorial methods *vs.* single ones, Bayesian statistics and transition analysis. Godde and Hens [41] found that the Bayesian approach outperformed the single method of Suchey-Brooks in the assessment of the pubic symphysis. Also in a comparison of transition analysis *vs.* traditional aging methods using a Mexican archaeological collection with individuals of unknown actual ages at death, Bullock et al. [42] found that the former more successfully captured older age estimates. Villa et al. [43] analysed the reliability of the Suchey-Brooks and Buckberry-Chamberlain methods using 3 D visualizations from CT and laser scans. While these methods can be improved with new imaging technology, currently direct observation from dry bone provides the most accurate results.

## Recent research

Published research in recent years has advanced age estimation methodology in diverse ways. New knowledge focuses on taphonomic impact, new anatomical areas yielding age information, histology, population variation, evaluation of the dental pulp chamber, technology, advanced mathematical approaches, biochemical analysis and age estimation of the living.

### Taphonomic impact

Age estimation methods are developed from anatomical collections containing individuals of known age at death. Such collections are usually in pristine condition, allowing examination of fragile structures. Of course, skeletons undergoing forensic anthropology analysis are rarely in pristine condition. Fragmented and taphonomically altered remains present unique challenges for age estimation.

While the negative impact of postmortem change has been known for decades, recent research has illuminated critical specific details. Cappella et al. [44] noted that preservation issues particularly impact the elderly. Their study of skeletal remains of 145 elderly individuals from Italy revealed that methods focusing on the fragile sternal ends of ribs and the palatine sutures were especially impacted. Likewise, as indicated by Adserias-Garriga et al. [45], thermal conditions severely affect the preservation of bone and teeth and thus limit age estimation.

### New anatomical areas of interest

Recent progress has been marked in the detection of new areas of the skeleton yielding age information. Alves-Cardoso and Assis [46] presented a detailed analysis of arthritic changes in joints of the skeleton and their correlation with age. Degenerative changes in the limb joints reflect age changes, in addition to habitual use and other factors. Such changes are variable but generally, correlate with advanced age. A similar, but more specific study focusing on just osteoarthritis of the shoulder presented useful data for older adults [47].

Also focusing on individuals of advanced age, Navega et al. [48] examined bone mineral density. Analysis of 100 female femora from the Coimbra, Portugal collection revealed a strong correlation between bone mineral density and age at death. Their methodological approach featured a connectionist computational approach and the use of artificial neural networks.

### Histology

Bone histology has been featured in approaches to age estimation since Kerley’s pioneering publication in 1965 [27] focusing on the midshaft of the femur, tibia and fibula. Subsequently, the approach has been modified for different structures and other bones of the skeleton. For decades, bone histology has been regarded as one of the most accurate individual techniques. However, its use by forensic anthropologists has been limited due to the specialized training required for specimen preparation and structure interpretation. A recent book edited by Crowder and Stout [49] reviews current applications of histology to anthropological issues. Streeter [50] provides a chapter summarizing recent histological aging research relating to different bones and anatomical areas within bones.

### Population variation

Forensic anthropologists must consider how population variation can affect the aging process. Such variation can reflect genetics but also socio-economic factors such as nutritional status. Recent research has documented much of this variation, primarily through regional testing of methods developed from other collections and locations. For example, Benedicto et al. [51] examined the validity and accuracy of three radiographic dental age estimation methods using a Brazilian sample of 387 males and 622 females ranging in age from 8 to 16 years. This study determined which of the existing methods were most applicable to Brazilian cases. In similar research, Guo et al. [52] presented Chinese data on second molar maturity using a large sample of 834 males and 823 females.

Sullivan et al. [53] recently added population data from a Western Australia sample with documented ages ranging from birth to 30 years on the timing of epiphyseal fusion in the pelvic girdle and proximal femur. In addition, working in Australia, Lottering et al. [54] provided new data on the timing of fusion of the iliac crest. The latter study examined 524 individuals ranging in age from 7 to 25 years using both conventional radiography and multislice computed tomography.

Methods to estimate age at death from the acetabulum are relatively new and thus have attracted recent research attention aimed at validation. Calce and Rogers [55] evaluated methods of age estimation using the acetabulum on a sample of 100 males from the documented Grant collection in Ontario, Canada. Their research demonstrated the value of consulting local reference samples in age estimation.

Herrera and Retamal [56] examined the reliability of age estimation from the iliac auricular surface using a documented Chilean sample. They found that in their sample, differences were difficult to detect between progressive age phases defined by Osborne et al. [57]. Michopoulou et al. [58] tested other methods of age estimation from the auricular surface finding a more significant error in their applications to documented skeletons from Crete, Greece than initially reported.

Working with a contemporary Mexican sample, Muñoz et al. [59] tested published age standards for the sternal end of the fourth rib. Their analysis of 444 males and 60 females with known ages at death ranging from 17 to 92 years revealed that the published standards underestimated age. In addition, they found that in males, inaccuracy increased with advancing age as had been commonly assumed previously.

Variation in the aging process occurs not only in individuals from different regions but also those of varying body sizes and socioeconomic levels. Merritt [60] tested eight methods of age estimation on 746 North American skeletons of varying body mass and living stature. The Merritt study revealed that body size does affect the accuracy of age estimation.

Spake and Cardoso [61] examined differences in age estimation using bone size between average children and victims of homicide. This study suggested that growth variation related to socioeconomic status impacted age estimation and revealed differences between data from the United States and Australia/New Zealand.

### Dental pulp chamber

Teeth provide useful age information, especially with subadults. Recent research indicates that the pulp chamber, the inner area that houses sensitive nerves, provides data useful for estimating age in adults [62]. Building on methodology developed by Cameriere et al. [63–67], D’Ortenzio et al. [68] examined age changes in the pulp/tooth ratio calculated from tooth sections. Their research samples included individuals of known age at death and offered a new method exceptionally accurate for age categories greater than 50 years.

More recently, Dehghani et al. [69] provided new data from Iran in an assessment of age changes in the dental pulp chamber of canines using digital panoramic radiographs. Using a large sample of maxillary and mandibular canines, they employed CAD software and regression analysis. Their research provided useful information on population variation and indicated that maxillary canines yield more accurate results than those from the mandible.

Roberts et al. [70] noted that assessment of root canal widths of mandibular molars can assist age estimation, especially regarding determination if the individual is above or below the age of 18 years. Their London, UK radiographic study utilized 1 000 males and 1 000 females of ages between 16 and 26 years. The attempt to determine if the age of an individual is above or below a specific age (e.g. 18 years) relates to medico-legal issues of the living. This process is distinct from the prediction of a likely age range in a paleodemographic study or the assessment of an unidentified skeleton. Note also that factors of selected mortality and related issues may affect the use of data from the living to predict age of the deceased.

### Technology and mathematics

With remarkable advancements in technology, forensic anthropology has taken old ways of estimating age and adapted them to be compatible with new equipment. These new approaches, such as 3 D scanning, often promise greater accuracy in estimating age at death. Considerable experimentation has focused on scanning the pubic symphysis. Stoyanova et al. [71] have addressed high subjectivity and error in assessing age using scans of the pubic symphysis. They propose combining different 3 D laser scans to layer on top of each other to produce an accurate image of the pubic symphysis.

Calce and Rogers [55] attempted to advance aging methods involving the acetabulum by assessing a myriad of its features. They concluded that their technique is most accurate for adults over the age of 40. Recently, research has explored if the acetabulum could be used to estimate age at death in adults through geometric morphometrics [72]. Geometric morphometrics is the analysis of coordinates in a geometric rather than a linear manner. Their research aimed to analyse age changes in the acetabulum for both males and females. Sex must be considered when estimating age from the acetabulum because of well-documented sexual dimorphism in size.

New mathematical approaches, such as multivariate adaptive regression splines [73], play an important role in estimating age at death. In 2018, Koterová et al. [74] reported their use of nine different mathematical approaches to assess accuracy. These calculations were focused on the pubic symphysis and the auricular surface. Although further research is required, results suggested that combining complex mathematical calculations would improve age estimation. Similar results were reported by Štepanovský et al. [75] in their study of 22 methods applied to a Czech sample of 622 males and 314 females between the ages of 2.7 and 20.5 years.

Although impressive new technology is now available, it is important to remember that sometimes conventional observation is better. Villa et al. [43] compared methods to estimate age from the pubic symphysis and auricular area of the pelvis using direct observation on the dry bones and 3 D visualizations from CT and laser scans. Since the methods tested were developed on dry bone, those observations offered better results than the technological visualizations. Potentially, new methods could be developed focused specifically on 3 D digital data.

### Biochemical analysis

Biochemistry, a branch of chemistry that analyses compounds within living organisms, provides useful tools in forensic science. In the scope of forensic anthropology, the biochemical analysis focusing extensively on DNA offers vital information on age at death [45,76–79].

Zapico and Ubelaker [80] assessed the varied applications of the physiological bases of the aging process to the estimation of age at death in human skeletal remains. They listed the following as top prospects for new methodological approaches in age estimation: aspartic acid racemization, lead accumulation, collagen cross-links, Ramon microspectrometry for dental composition, advanced glycation end products (AGEs), telomere shortening, mitochondrial mutations and the decline of sjTREC rearrangements. Specific studies of mitochondrial DNA mutations in dentin and pulp of teeth in Spanish samples revealed promising results [81].

Use of a methylation marker set represents a very promising molecular approach to age estimation [82]. Methylation levels in selected loci from blood samples allowed age to be predicted with a median error of only 3.07 years.

## Estimating age of the living

While this article predominately focuses on skeletal remains of deceased individuals, some forensic cases call for estimating the age of the living. Such cases relate to legal issues associated with migration, child pornography, juvenile/adult status of those accused of crimes and age progression of missing persons [83]. Many of these issues involve country-specific definitions of adult legal status concerning individuals of undocumented age.

Much recent progress on the methodology of age estimation of the living stems from efforts of the Study Group on Forensic Age Diagnostics (AGFAD) founded in Berlin, Germany in March 2000 [84]. This thoughtful group examined the criteria for age estimation, including ethics and the need to address the specific problem presented for resolution. Recommended methodology focused on the physical examination, radiography of the left hand and dental examination, including x-rays or related imagery. Following these recommendations, Guo et al. [85] noted that the extent of the epiphyseal union of the clavicles also provides important information. They provided key data through a magnetic resonance imaging study of 269 males and 248 females from Germany with ages from 12 to 24 years. The resulting methodology has been tested in Turkey using CT scan data with positive results [86]. Schmeling et al. [87] provide a summary of the methodological approaches and the related legal issues.

Recently, Matthews et al. [88] published an approach to estimate age and to predict patterns of growth using 3 D facial prototypes. Their study focused on children and adolescents using a sample of 422 boys and 442 girls of Australian, European or North American ethnicity. A subset of 24 boys and 26 girls participated in a longitudinal validation study. The resulting data and method should prove useful in both age estimation and predicting growth from photographs.

Estimation of age in the living continues to be challenging, primarily due to the limited visible data available, ethical issues related to radiographic and intrusive techniques and the complexity introduced by population variation. Such estimation is especially difficult when the ancestral origins and nutritional history of the individual are unknown.

## Summary and future directions

Historically, estimation of age at death of human skeletal remains has represented a central forensic anthropological activity. In recent years, research has focused extensively on improving methodology and understanding the many related issues. Casework and recent research have clarified the powerful impact of postmortem change on age estimation. Some of the most accurate and dependable methods are not available for incomplete skeletons and those showing destruction of fragile and vulnerable elements.

Histological approaches are now available for different areas of the skeleton and different structures. Appropriate training and availability of the necessary equipment for this destructive technique remain challenging.

Many studies now document the population variation involved in the aging process. Fortunately, new collections and research data from many areas of the world have materialized. Increasingly, local standards are available for consultation and the errors involved in more general applications are better understood. Consideration also must be given to body size and socioeconomic status within regions and nutrition.

Recent research demonstrates the value of dental pulp chamber evaluation in age estimation. Advances include histological sectioning and direct measurement of root canal size.

Clearly, advances in technology and mathematical approaches in analysis impact age estimation. The 3 D imagery and scanning procedures offer opportunities to gather data from the living and to examine skeletal structure in fully fleshed or partially decomposed decedents. Rigorous testing indicates that at times, direct examination of dry bone represents the method of choice and consideration of multiple mathematical approaches is desirable. Clearly, as in other areas of forensic science, technological advancement likely will continue to fuel progress in anthropology. In addition to imagery, the technology and associated knowledge relating to biochemical analysis show great promise for advancement in age estimation, although taphonomic factors remain a concern.

Recent literature indicates that skeletal remains continue to be a central focus of age estimation methodology and practice as suggested by the Scientific Working Group for Forensic Anthropology (SWGANTH) document on Age Estimation [89]. However, age estimation of the living has emerged as a significant application as well. Legal issues relating to migration, adoption, child pornography, criminal behavior and missing persons all call for an accurate assessment of age. Progress on age estimation of the living is closely related with technological advancement since direct observation of dry bone is not an option. Age estimation of the living is problematic and must consider important legal and ethical issues.

Future progress in age estimation methodology closely links with growing global interest in the general field of forensic anthropology. Increasing numbers of well-qualified, trained and highly motivated forensic anthropologists in many countries likely will lead to more documented collections of human remains, increased careful experimentation and research, and growing publication opportunities. Such developments will stimulate future progress in the process of age estimation.

## References

[1] Blau S, Ubelaker DH, editors. Handbook of forensic anthropology and archaeology. New York City (NY): Routledge; 2016.

[2] Christensen AM, Passalacqua NV, Bartelink EJ Forensic anthropology: current methods and practice. San Diego (CA): Elsevier Inc; 2014.

[3] Latham KE, Finnegan M, editors. Age estimation of the human skeleton. Springfield (IL): Charles C. Thomas; 2010.

[4] Ubelaker DH Human skeletal remains: Excavation, analysis, interpretation. Washington (DC): Taraxacum; 1999.

[5] Rogers TL Skeletal age estimation In: Blau S, Ubelaker DH, editors. Handbook of forensic anthropology and archaeology. New York City (NY): Routledge; 2016; p. 273–292.

[6] Brooks ST Skeletal age at death: the reliability of cranial and pubic ageindicators. Am J Phys Anthropol. 1955;13:567–597. 13292526 10.1002/ajpa.1330130403

[7] Krogman WM The human skeleton in forensic medicine. Springfield (IL): Charles C. Thomas; 1962.

[8] Stewart TD Essentials of forensic anthropology: especially as developed in the United States. Springfield (IL): Charles C. Thomas; 1979.

[9] Bocquet-Appel JP, Masset C Farewell to paleodemography. J Hum Evol. 1982;11:321–333.

[10] Van Gerven DP, Armelagos GJ “Farewell to paleodemography?” rumors of its death have been greatly exaggerated. J Hum Evol. 1983;12:353–360.

[11] Bocquet-Appel JP, Masset C Paleodemography: Expectancy and false hope. Am J Phys Anthropol. 1996;99:571–583. 8779339 10.1002/(SICI)1096-8644(199604)99:4<571::AID-AJPA4>3.0.CO;2-X

[12] Meindl RS, Lovejoy CO, Mensforth RP Skeletal age at death: accuracy of determination and implications for human demography. Hum Biol. 1983;55:73–87. 6840749

[13] Meindl RS, Lovejoy CO Ectocranial suture closure: a revised method for the determination of skeletal age at death based on the lateral-anterior sutures. Am J Phys Anthropol. 1985;68:57–66. 4061602 10.1002/ajpa.1330680106

[14] Meindl RS, Lovejoy CO, Mensforth RP, Walker RA A revised method of age determination using the os pubis, with a review and tests of accuracy of other current methods of pubic symphyseal aging. Am J Phys Anthropol. 1985;68:29–45. 4061600 10.1002/ajpa.1330680104

[15] Lovejoy CO, Meindl RS, Pryzbeck TR, et al. Chronological metamorphosis of the auricular surface of the ilium: a new method for the determination of adult skeletal age at death. Am J Phys Anthropol. 1985;68:15–28. 4061599 10.1002/ajpa.1330680103

[16] Lovejoy CO, Meindl RS, Mensforth RP, et al. Multifactorial determination of skeletal age at death: a method and blind tests of its accuracy. Am J Phys Anthropol. 1985;68:1–14. 4061595 10.1002/ajpa.1330680102

[17] Brooks S, Suchey JM Skeletal age determination based on the os pubis: a comparison of the Acsádi-Nemeskéri and Suchey-Brooks methods. J Hum Evol. 1990;5:227–238.

[18] Konigsberg LW, Frankenberg SR Deconstructing death in paleodemography. Am J Phys Anthropol. 2002;117:297–309. 11920365 10.1002/ajpa.10039

[19] Bedford ME, Russell KF, Lovejoy CO, et al. Test of the multifactorial aging method using skeletons with known ages-at-death from the Grant collection. Am J Phys Anthropol. 1993;91:287–297. 8333486 10.1002/ajpa.1330910304

[20] Hershkovitz I, Latimer B, Dutour O, et al. Why do we fail in aging the skull from the sagittal suture? Am J Phys Anthropol. 1997;103:393–399. 9261501 10.1002/(SICI)1096-8644(199707)103:3<393::AID-AJPA8>3.0.CO;2-R

[21] Aykroyd RG, Lucy D, Pollard AM, et al. Technical note: regression analysis in adult age estimation. Am J Phys Anthropol. 1997;104:259–265. 9386831 10.1002/(SICI)1096-8644(199710)104:2<259::AID-AJPA11>3.0.CO;2-Z

[22] Belkin V, Livshits V, Otremski I, et al. Aging bone score and climatic factors. Am J Phys Anthropol. 1998;106:349–359. 9696150 10.1002/(SICI)1096-8644(199807)106:3<349::AID-AJPA7>3.0.CO;2-H

[23] Galera V, Ubelaker DH, Hayek L Comparison of macroscopic cranial methods of age estimation applies to skeletons from the Terry collection. J Forensic Sci. 1998;43:933–939. 9729807

[24] Baccino E, Ubelaker DH, Hayek L, et al. Evaluation of seven methods of estimating age at death from mature human skeletal remains. J Forensic Sci. 1999;44:931–936. 10486944

[25] Lamendin H, Baccino E, Humbert JF, et al. A simple technique for age estimation in adult corpses: the two criteria dental method. J Forensic Sci. 1992;37:1373–1379. 1402761

[26] Íşcan MY, Loth SR, Wright RK Age estimation from the rib by phase analysis: white males. J Forensic Sci. 1984; 65:147–156. 6502109

[27] Kerley ER The microscopic determination of age in human bone. Am J Phys Anthropol. 1965;23:149–163. 5826273 10.1002/ajpa.1330230215

[28] Milner GR, Wood JW, Boldsen JL Paleodemography In: Katzenberg MA, Saunders SR, editors. Biological anthropology of the human skeleton. Hoboken (NJ): John Wiley & Sons. Inc; 2000; p. 467–497.

[29] Hoppa RD Population variation in osteological aging criteria: an example from the pubic symphysis. Am J Phys Anthropol. 2000;111:185–191. 10640946 10.1002/(SICI)1096-8644(200002)111:2<185::AID-AJPA5>3.0.CO;2-4

[30] Wittwer-Backofen U, Buba H Age estimation by tooth cementum annulation: perspectives of a new validation study In: Hoppa RD, Vaupel JW, editors. Paleodemography: age distributions from skeletal samples. Cambridge (UK): Cambridge University Press; 2002 p. 107–128.

[31] Boldsen JL, Milner GR, Konigsberg LW, et al. Transition analysis: a new method for estimating age from skeletons In: Hoppa RD, Vaupel JW, editors. Paleodemography: Age distributions from skeletal samples. Cambridge (UK): Cambridge University Press; 2002 p. 73–106.

[32] Kemkes-Grottenthaler A Aging through the ages: Historical perspectives on age indicator methods In: Hoppa RD, Vaupel JW, editors. Paleodemography: Age distributions from skeletal samples. Cambridge (UK): Cambridge University Press; 2002 p. 48–72.

[33] Hoppa RD, Vaupel JW The Rostock Manifesto for paleodemography: The way from stage to age In: Hoppa RD, Vaupel JW, editors. Paleodemography: Age distributions from skeletal samples. Cambridge (UK): Cambridge University Press; 2002 p. 1–8.

[34] Bello SM, Thomann A, Signoli M, et al. Age and sex bias in the reconstruction of past population structures. Am J Phys Anthropol. 2006;129:24–38. 16161146 10.1002/ajpa.20243

[35] Rinaldi A, Pacciani E, Gori S [Cranial suture closure as age-at-death indicator: a further test of its reliability] In: Di Bacco M, Frederic P, Scallfari F, editors. [Bayesian inference for diagnosing sex and age-at-death in skeletal remains]. Asti: Quaderni di Asti Studi Superiori; 2006 p. 41–46. Italian.

[36] Frederic P, Pacciani E, Gori S [Age-at-death diagnosis by tooth wear: reliability evaluation]. A Bayesian model In: Di Bacco M, Frederic P, Scallfari F, editors. [Bayesian inference for diagnosing sex and age-at-death in skeletal remains]. Asti: Quaderni di Asti Studi Superiori; 2006 p. 19–29. Italian.

[37] Ardito V, Pacciani E [Bayesian inference on age-at-death and age-at-death distribution from a continuous age indicator] In: Di Bacco M, Frederic P, Scallfari F, editors. [Bayesian inference for diagnosing sex and age-at-death in skeletal remains]. Asti: Quaderni di Asti Studi Superiori; 2006 p. 1–17. Italian.

[38] Milner GR, Wood JW, Boldsen JL Advances in paleodemography In: Katzenberg MA, Suanders SR, editors. Biological anthropology of the human skeleton. 2nd edition Hoboken (NJ): John Wiley & Sons. Inc; 2008 p. 561–600.

[39] Garvin HM, Passalacqua NV, Uhl NM, et al. Developments in forensic anthropology: Age-at-death estimation In: Dirkmaat DC, editor. A companion to forensic anthropology. West Sussex: Blackwell Publishing Ltd; 2012 p. 203–223.

[40] Milner GR, Boldsen JL Skeletal age estimation: Where we are and where we should go In: Dirkmaat DC, editor. A companion to forensic anthropology. [West Sussex]: Blackwell Publishing Ltd; 2012 p. 224–238.

[41] Godde K, Hens SM Age-at-death estimation in an Italian historical sample: a test of the Suchey-Brooks and transition analysis methods. Am J Phys Anthropol. 2012;149:259–265. 22886637 10.1002/ajpa.22126

[42] Bullock M, Márquez L, Hernández P, et al. Paleodemographic age-at-death distributions of two Mexican skeletal collections: a comparison of transition analysis and traditional aging methods. Am J Phys Anthropol. 2013;152:67–78. 24037796 10.1002/ajpa.22329

[43] Villa C, Buckberry J, Cattaneo C, et al Technical note: reliability of Suchey-Brooks and Buckberry-Chamberlain methods on 3D visualizations from CT and laser scans. Am J Phys Anthropol. 2013;151:158–163. 23595646 10.1002/ajpa.22254

[44] Cappella A, Cummaudo M, Arrigoni E, et al. The issue of age estimation in a modern skeletal population: are even the more modern current aging methods satisfactory for the elderly? J Forensic Sci. 2017;62:12–17. 27783413 10.1111/1556-4029.13220

[45] Adserias-Garriga J, Thomas C, Ubelaker DH, et al. When forensic odontology met biochemistry: Multidisciplinary approach in forensic human identification. Arch Oral Biol. 2018;87:7–14. 29241027 10.1016/j.archoralbio.2017.12.001

[46] Alves-Cardoso F, Assis S Can osteophytes be used as age at death estimators? Testing correlations in skeletonized human remains with known age-at-death. Forensic Sci Int. 2018;288:59–66. 29729497 10.1016/j.forsciint.2018.04.034

[47] Brennaman AL, Love KR, Bethard JD, et al. A Bayesian approach to age-at-death estimation from osteoarthritis of the shoulder in modern North Americans. J Forensic Sci. 2017;62:573–584. 27930820 10.1111/1556-4029.13327

[48] Navega D, Coelho J, Cunha E, et al. DXAGE: a new method for age at death estimation based on femoral bone mineral density and artificial neural networks. J Forensic Sci. 2018;63:497–503. 28851106 10.1111/1556-4029.13582

[49] Crowder C, Stout S Bone histology – an anthropological perspective. Boca Raton (FL): Taylor & Francis Group, LLC; 2012.

[50] Streeter M Histological age-at-death estimation In: Crowder C, Stout S editors. Bone histology – An anthropological perspective. Boca Raton (FL): CRC Press; 2012 p. 135–152.

[51] Benedicto EN, Azevedo ACS, Michel-Crosato E, Biazevic MGH Validity and accuracy of three radiographic dental age estimation methods in Brazilians. Forensic Sci Int. 2018; 283:128–135. 29301112 10.1016/j.forsciint.2017.12.014

[52] Guo Y, Chu G, Olze A, et al. Age estimation of Chinese children based on second molar maturity. Int J Legal Med. 2018;132:807–813. 29034417 10.1007/s00414-017-1703-6

[53] Sullivan S, Flavel A, Franklin D Age estimation in a sub-adult Western Australian population based on the analysis of the pelvic gridle and proximal femur. Forensic Sci Int. 2017;281:185e1–185e10. 29108763 10.1016/j.forsciint.2017.10.010

[54] Lottering N, Alston-Knox CL, MacGregor DM, et al. Apophyseal ossification of the iliac crest in forensic age estimation: computed tomography standards for modern Australian subadults. J Forensic Sci. 2017;62:292–307. 27885641 10.1111/1556-4029.13285

[55] Calce SE, Rogers TL Evaluation of age estimation technique: testing traits of the acetabulum to estimate age at death in adult males. J Forensic Sci. 2011;56:302–311. 21306380 10.1111/j.1556-4029.2011.01700.x

[56] Herrera MJ, Retamal R Reliability of age estimation from iliac auricular surface in a subactual Chilean sample. Forensic Sci Int. 2017;275:317e1–317e4. 28314517 10.1016/j.forsciint.2017.01.029

[57] Osborne DL, Simmons TL, Nawrocki SP Reconsidering the auricular surface as an indicator of age at death. J Forensic Sci. 2004;4:905–911. 15461088

[58] Michopoulou E, Negre P, Nikita E, et al. The auricular surface as age indicator in a modern Greek sample: a test of two qualitative methods. Forensic Sci Int. 2017;280:246e1–246e7. 28965664 10.1016/j.forsciint.2017.08.004

[59] Muñoz A, Maestro N, Benito M, et al. Sex and age at death estimation from the sternal end of the fourth rib. Does Íşcan's method really work? Leg Med (Tokyo). 2018;31:24–29. 29272755 10.1016/j.legalmed.2017.12.002

[60] Merritt CE Inaccuracy and bias in adult skeletal age estimation: assessing the reliability of eight methods on individuals of varying body sizes. Forensic Sci Int. 2017;275:315e1–315e11. 28359575 10.1016/j.forsciint.2017.03.003

[61] Spake L, Cardoso HFV Are we using the appropriate reference samples to develop juvenile age estimation methods based on bone size? An exploration of growth differences between average children and those who become victims of homicide. Forensic Sci Int. 2018; 282:1–12. 29136574 10.1016/j.forsciint.2017.10.041

[62] Marroquin TY, Karkhanis S, Kvaal SI, et al. Age estimation in adults by dental imaging assessment systematic review. Forensic Sci Int. 2017;275:203–211. 28410514 10.1016/j.forsciint.2017.03.007

[63] Cameriere R, Ferrante L, Cingolani M Variations in pulp/tooth area ratio as an indicator of age: a preliminary study. J Forensic Sci. 2004;49:317–319. 15027553

[64] Cameriere R, Ferrante L, Belcastro MG, et al Age estimation by pulp/tooth ratio in canines by mesial and vestibular peri-apical X-rays. J Forensic Sci. 2007;52:1151–1155. 17680998 10.1111/j.1556-4029.2007.00530.x

[65] Cameriere R, Cunha E, Sassaroli E, et al. Age estimation by pulp/tooth area ratio in canines: Study of a Portuguese sample to test Cameriere’s method. Forensic Sci Int. 2009;193:128e1–128e6. 19854595 10.1016/j.forsciint.2009.09.011

[66] Cameriere R, De Luca S, Alemán I, et al. Age estimation by pulp/tooth ratio in lower premolars by orthopantomography. Forensic Sci Int. 2012;214:105–112. 21821373 10.1016/j.forsciint.2011.07.028

[67] Cameriere R, Cunha E, Wasterlain SN, et al. Age estimation by pulp/tooth ratio in lateral and central incisors by peri-apical X-ray. J Forensic Leg Med. 2013;20:530–536. 23756528 10.1016/j.jflm.2013.02.012

[68] D'Ortenzio L, Prowse T, Inskip M, et al. Age estimation in older adults: use of pulp/tooth ratios calculated from tooth sections. Am J Phys Anthropol. 2018;165:594–603. 29238950 10.1002/ajpa.23371

[69] Dehghani M, Shadkam E, Ahrari F, et al. Age estimation by canines’ pulp/tooth ratio in an Iranian population using digital panoramic radiography. Forensic Sci Int. 2018;285:45–49. 10.1016/j.forsciint.2018.01.016 29433010

[70] Roberts GJ, Lucas VS, Andiappan M, et al. Dental age estimation: pattern recognition of root canal widths of mandibular molars. A novel mandibular maturity marker at the 18-year threshold. J Forensic Sci. 2017;62:351–354. 27907239 10.1111/1556-4029.13287

[71] Stoyanova DK, Algee-Hewitt BFB, Kim J, et al. A computational framework for age-at-death estimation from the skeleton: surface and outline analysis of 3D laser scans of the adult pubic symphysis. J Forensic Sci. 2017;62:1434–1444. 28244105 10.1111/1556-4029.13439

[72] San-Millán M, Rissech C, Turbón D Shape variability of the adult human acetabulum and acetabular fossa related to sex and age by geometric morphometrics. Implications for adult age estimation. Forensic Sci Int. 2017;272:50–63. 28113134 10.1016/j.forsciint.2017.01.005

[73] Corron L, Marchal F, Condemi S, et al. A new approach of juvenile age estimation using measurements of the ilium and multivariate adaptive regression splines (MARS) models for better age predication. J Forensic Sci. 2017;62:18–29. 27792240 10.1111/1556-4029.13224

[74] Kotěrová A, Navega D, Štepanovský M, et al. Age estimation of adult human remains from hip bones using advanced methods. Forensic Sci Int. 2018;287:163–175. 29674227 10.1016/j.forsciint.2018.03.047

[75] Štepanovský M, Ibrová A, Buk Z, et al. Novel age estimation model based on development of permanent teeth compared with classical approach and other modern data mining methods. Forensic Sci Int. 2017;279:72–82. 28850870 10.1016/j.forsciint.2017.08.005

[76] Carracedo A From hemogenetic to forensic genomics In: Ferrara SD, editor. P5 medicine and justice – innovation, unitariness and evidence. Cham: Springer International Publishing AG; 2017 p. 439–447.

[77] Cassina M, Clementi M DNA-based methods for age estimation In: Ferrara SD, editor. P5 medicine and justice – innovation, unitariness and evidence. Cham: Springer International Publishing AG; 2017 p. 413–425.

[78] Lacan M, Thèves C, Amory S, et al. Detection of the A189G mtDNA heteroplasmic mutation in relation to age in modern and ancient bones. Int J Legal Med. 2009;123:161–167. 18622623 10.1007/s00414-008-0266-y

[79] Papiha SS, Rathod H, Briceno I, et al. Age related somatic mitochondrial DNA deletions in bone. J Clin Pathol. 1998;51:117–120. 9602684 10.1136/jcp.51.2.117 PMC500505

[80] Zapico SC, Ubelaker DH Applications of physiological bases of ageing to forensic sciences. Estimation of age-at-death. Aging Res Rev. 2013;12:605–617. 10.1016/j.arr.2013.02.002 23454111

[81] Zapico SC, Ubelaker DH Relationship between mitochondrial DNA mutations and aging. Estimation of age-at-death. J Gerontolog A Biol Sci Med Sci. 2016;71:445–450. 10.1093/gerona/glv115 26286606

[82] Freire-Aradas A, Phillips C, Mosquera-Miguel A, et al. Development of a methylation marker set for forensic age estimation using analysis of public methylation data and the Agena Bioscience EpiTYPER system. Forensic Sci Int. 2016;24:65–74. 10.1016/j.fsigen.2016.06.005 27337627

[83] Black S, Aggrawal A, Payne-James J Editors. Age estimation in the living: the Practitioner’s Guide. West Sussex: Wiley-Blackwell; 2010.

[84] Schmeling A, Geserick G, Reisinger W, et al. Age estimation. Forensic Sci Int. 2007;165:178–181. 16782291 10.1016/j.forsciint.2006.05.016

[85] Guo Y, Olze A, Ottow C, et al. Dental age estimation in living individuals using 3.0 T MRI of lower third molars. Int J Legal Med. 2015;129:1265–1270. 26232290 10.1007/s00414-015-1238-7

[86] Ekizoglu O, Hocaoglu E, Inci E, et al. Forensic age estimation by the Schmeling method: computed tomography analysis of the medial clavicular epiphysis. Int J Legal Med. 2015;129:203–210. 10.1007/s00414-014-1121-y 25408292

[87] Schmeling A, Dettmeyer R, Rudolf E, et al. Forensic age estimation – methods, certainty, and the law. Dtsch Arztebl Int. 2016;113:44–50. 26883413 10.3238/arztebl.2016.0044 PMC4760148

[88] Mathews H, Penington A, Clement J, et al. Estimating age and synthesizing growth in children and adolescents using 3D facial prototypes. Forensic Sci Int. 2018;286:61–69. 29567544 10.1016/j.forsciint.2018.02.024

[89] Scientific Working Group for Forensic Anthropology (SWGANTH) Age Estimation. 2013; Available from: https://www.nist.gov/sites/default/files/documents/2018/03/13/swganth_age_estimation.pdf

